# Seasonal impact of acid mine drainage on water quality and potential ecological risk in an old sulfide exploitation

**DOI:** 10.1007/s11356-024-32367-1

**Published:** 2024-02-22

**Authors:** Patrícia Gomes, Teresa Valente

**Affiliations:** Institute of Earth Sciences, Pole of University of Minho, University of Minho, Campus de Gualtar, 4710-057 Braga, Portugal

**Keywords:** Acid mine drainage, Seasonal variability, Water environmental quality, Potential ecological risk index, Drought episodes

## Abstract

Sulfides are usually associated with deposits of metals and coal. The reactive wastes from their exploitation, typically stored in piles and tailings dams, are often the mining sector’s primary source of environmental problems. The surrounding river waters can present signs of acid mine drainage, responsible for aquatic ecosystem degradation. So, the main target of the present study is to investigate the impact of this process on the water’s environmental quality and potential ecological risk. The study area is located at the Iberian Pyrite Belt, in an old sulfide exploitation, closed without environmental rehabilitation measures. The results exhibit high sulfate concentrations (410,601 mg/L) and potentially toxic elements, with prominence of Fe (134,000 mg/L), overcoming many other extreme cases of AMD pollution. The Ficklin diagram exposes that most samples are classified as “high-acid, high-metal.” Two of them have extreme classifications (high-acid, extreme-metal). The pH value is well below the acceptable range for the environmental quality of superficial waters (5–7), measuring at a minimum of 0.84. Regarding seasonal variability, the study showed a higher degree of contamination in dry conditions (e.g., 4,420 mg/L of Cu), while the rainy month had lower concentrations of PTE (186.8 mg/L of Cu for the same sampling point). In addition, the water does not accomplish the environmental objectives established by the EU Water Framework Directive. According to the new approach developed based on a scale adjustment, the potential ecological risk index studied indicates that most sampled sites present strong, very strong, and even extremely potential ecological risk. With a typical Mediterranean climate, the region suffers from water scarcity, predicting increasingly in the future more degrading scenarios for water environmental quality. Consequently, urgent mitigation and remediation measures are necessary to improve and preserve water quality and fulfill the objectives of the United Nations Sustainability Development Goals.

## Introduction

Sulfides are the most common minerals, stable under reducing circumstances (Nordstrom and Alpers 1999). Lottermoser (2010) highlights the role of pyrite, marcasite, pyrrhotite, and chalcopyrite due to their abundance and environmental relevance under oxidative dissolution conditions. They are usually associated with coal and metallic mining (e.g., Cu, Zn, and Pb). However, exploitation of these elements can generate large amounts of waste that are typically stored in specific infrastructures, such as piles around the mine and tailings dams, constituting the main environmental focus of the mining sector (e.g., Sánchez España et al. 2008). The problem centers on mineral–water, mineral-atmosphere, and mineral-biosphere interactions, producing acid mine drainage (AMD) (Nordstrom et al. 2015). Classic mining landscapes start to present waters with typical ochre coloration, supergenic minerals in various bright colors (Alpers et al. 1994; Wolkersdorfer et al. 2020), and crustification of streambeds (Valente et al. 2012). Also, the receiving water systems are characterized by low pH, high concentrations of acidity, sulfate, and potentially toxic elements (PTE) (Gomes et al. 2018), which often behave as permanent pollutants (Kicińska et al. 2021). From an ecological point of view, the specific impact of sulfide-rich wastes is often manifested by the appearance of extremophiles, where acidophilic algae and other microorganisms essentially proliferate (e.g., Aguilera et al. 2006; Amils et al. 2011; Levings et al. 2005; Schneider et al. 2018; Gomes et al. 2021).

As water is an essential resource for the maintenance of biota, its environmental quality is a critical factor influencing ecosystems and human health (Zhang et al. 2012). Therefore, water availability with a suitable value for specific uses, like human consumption or maintenance of ecological quality, is a crucial issue, especially in semi-arid climates (Tiri et al. 2014). Nevertheless, water scarcity associated with frequent drought generates deep concern about the quantity and quality of this vital resource (Bonnail et al. 2019). According to the same authors, the environmental quality standards in aquatic ecosystems have been increasingly prioritized by the EU Water Framework Directive (WFD, Directive, 2000/60/EU, and modifications such as Directives, 2008/105/EU and 2013/39/EU). “Good chemical status,” such as PTE, and “ecological status” are two fundamental environmental quality standards for surface waters. According to Zhou et al. (2022) and Zhang et al. (2019), it is necessary to identify factors, such as land use, meteorology, and hydrology, that may contribute to degradation and explore their effects on water quality to protect riverine water resources scientifically. In this context, according to knowledge, no study has been carried out that allows the assessment of surface waters in the old mining area of São Domingos about current EU requirements. Furthermore, it developed and proposed an addition of new classes of potential ecological risk index. So, this work intends to investigate the fulfillment of quality environmental objectives for surface water, the impact that water scarcity, related to seasonality, may have in the aquatic medium, and the potential ecological risk inherent in current conditions. The present study focuses on the hydrochemical properties of two streams that run through an old sulfide mine, representing a unique scenario of AMD contamination. Downstream, the reservoir (The Chança River) is used for drinking water production. So, the study contemplated 12 sampling points, including a pit lake and water dams along the streams, over a complete hydrological year. Thus, this investigation intends to contribute to the knowledge about the available water bodies, warning to their appropriate management — in terms of improving ecological and functional quality — in a traditional mining region with a typical Mediterranean climate that faces water shortages.

## Materials and methods

### Study area and sampling sites

**Fig. 1:**
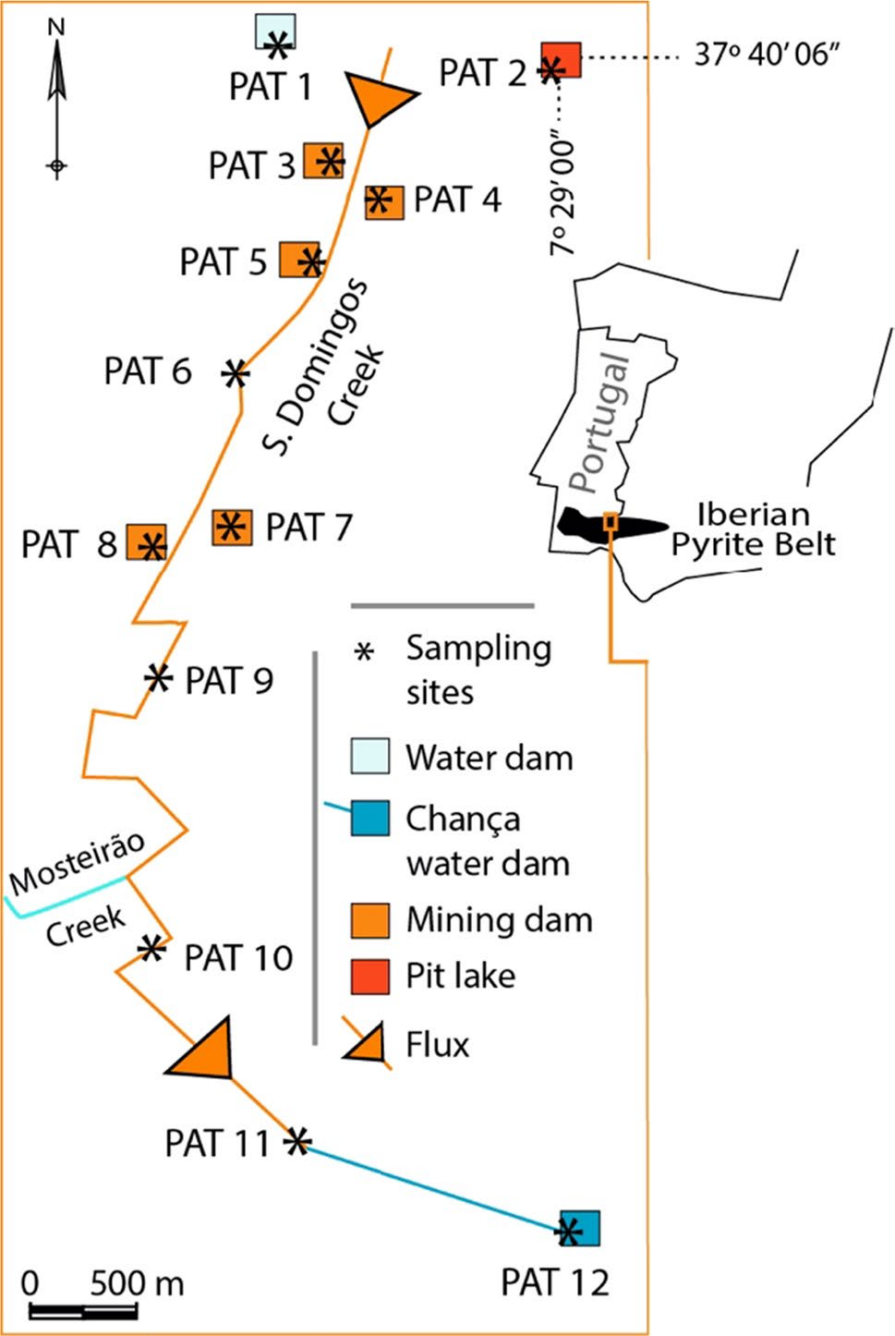
Sketch with the location of the São Domingos mine in the Portuguese sector of the Iberian Pyrite Belt and respective sampling sites (PAT1 to PAT12). Adapted from Gomes et al. 2022

**Fig. 2:**
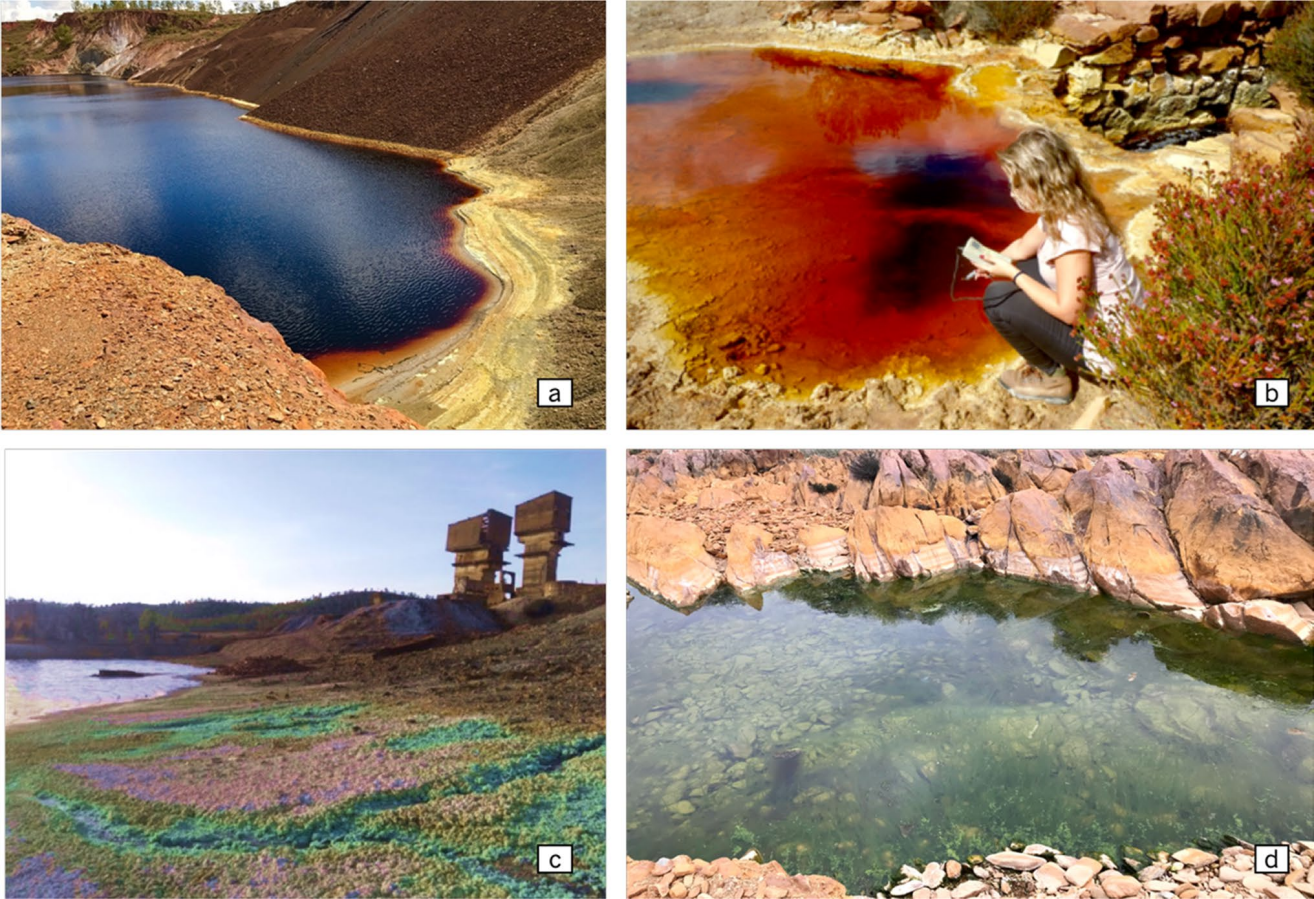
Field images illustrating some sampling points: **a** PAT2 (pit lake); **b** PAT6 (typical ochre color of acid mine drainage water); **c** PAT7 (lentic environment with efflorescent salts); **d** PAT11 (lotic environment after confluence with Mosteirão river)

The Iberian Pyrite Belt (IPB) is a large metallogenic province in Portugal and Spain known as one of the major in the world (Fig. 1). Mining activities in the region have resulted in important contamination due to the exploitation of sulfide deposits. The São Domingos mine (Fig. 1), situated in the Portuguese sector of IPB, has a long history of mining activity dating back to pre-Roman times, with operations continuing until 1966. It was closed without environmental remediation, having recently started a rehabilitation project (EDM 2021; www.edm.pt.). Several authors (e.g., Abreu et al. 2008; Tavares et al. 2009; Pérez-López et al. 2008) characterized the study area as exhibiting low pH and abundant elements with potential implications for the environment and human health, such as Fe, Cu, Zn, Sb, As, Hg, and Pb. Numerous old infrastructures (ore-processing plants and machinery), waste dumps, and tailings dams are disseminated along the mining complex (Cordeiro et al. 2017). The pit lake (PAT2 in Figs. 1 and 2) reached a depth of 120 m and a perimeter of 2 km. PAT1, the Tapada Grande, was initially constructed for mining activities and is now a reservoir for recreational use. The remaining sampling points are located within the mining complex along the two main streams that drain the area: São Domingos and Mosteirão (Fig. 1). The waters have an intense red–orange color associated with high iron concentrations and ochre-precipitates deposition (Fig. 2a, b) (Gomes et al. 2017). Regarding aquatic biodiversity, blooms of acidophilic algae are frequently reported (Fig. 2d) (Wolowski et al. 2008; Luís et al. 2019) but with low diversity (Gomes and Valente 2019).

Samples were collected monthly from October 2016 to September 2017 in a complete hydrological year. The sampling sites can be categorized according to their hydrological conditions related to the flow regime. PAT1, PAT2, PAT3, PAT4, PAT5, PAT7, and PAT12 generally indicate lentic behavior, while the remaining PATs indicate lotic conditions (Fig. 2) (Gomes et al. 2022). The final receptor of the acidic discharges is the Chança River, here entitled with PAT12, which presently works as a reservoir for human supply.

### Climate conditions

**Fig. 3:**
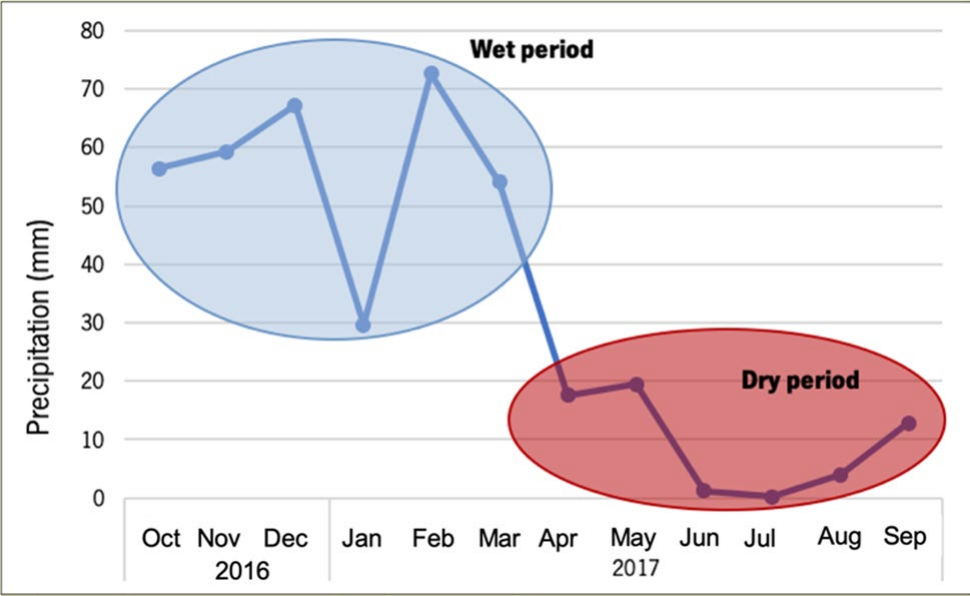
Monthly values of total precipitation, covering October 2016 to September 2017

Concerning average monthly precipitation, the 30 hydrological years (1936/37–1966/67) demonstrated that precipitation varied between 1.1 and 85.9 mm, in July and January, respectively. For the same period, the average air temperature varied between 10.4 °C in January and 25.7 °C in July, with the hottest period occurring from May to October, while November to April is the coldest period. According to the United Nations Framework Convention on Climate Change (ENAAC, 2010), Portugal is one of the European countries that is most vulnerable to the impacts of climate change. In this sense, risk situations such as precipitation peaks, heat waves, and storms associated with strong winds can occur more frequently (APA, 2016). The year of the present sampling was, especially in Portugal, atypical of high temperatures. The country suffered heat waves that led to fires of magnitudes and proportions never before reported, affecting numerous populations. Thus, the period corresponding to the sampling (2016 to 2018) was analyzed. So, the average monthly temperature for this period reveals that the driest months are also the hottest. The coldest month is January, with the lowest temperature recorded (7.7 °C) in 2017. July 2016 was the hottest month, with an average temperature of 26.6 °C. These data demonstrate higher thermal amplitude and temperature increase. Figure 3 presents the rainy season from October to March, with an average rainfall of 54 to 72 mm, and the dry period comprises the months between April and September, with rainfall ranging from 17.6 to 0.2 mm. Figure 3 also shows February as having the highest rainfall, 72.6 mm. July is the driest month, with 0.2 mm, showing less precipitation during the study period.

### Analytical methods

The pH, electrical conductivity (EC at 25 °C), and potential redox (Eh) were recorded in situ with a portable meter Thermo Scientific Orion. Surface water was collected for further analyses: 500 mL was used for sulfate determination, and 100 mL was filtered (0.45 µm) and acidified with nitric acid (75%) to maintain a pH below 2. The samples were immediately transported to the laboratory in polyethylene containers and refrigerated conditions (4 °C).

Sulfate was determined by turbidimetric method (Standard Methods 4500 E; APHA 2012). Selected element concentrations (Al, As, Cu, Fe, Ni, Co, Zn, Pb, and Cd) were obtained by inductively coupled plasma optical or mass spectrometry (ICP-OES/MS). These analyses were performed by Activation Laboratory, Lda—Actlabs, Canada, including duplicate samples and blanks to check precision, whereas accuracy was obtained by using certified standards (IV-STOCK-1643 (ICP/MS) Cert). All the reagents used were of analytical grade or Suprapur quality (Merck, Darmstadt, Germany). The standard solution was the Merck AA Certificate. Milli-Q water was utilized in all the experiments.

The SPSS Release 25.0 software was used to treat the results statistically. Ficklin diagram (Ficklin et al. 1992) was applied to distinguish and classify the different water samples.

### Potential ecological risk — a new approach

According to different authors (e.g., Ojekunle et al. 2016; Withanachchi et al. 2018), the metal index (MI) and the potential ecological risk index (RI) provide the bigger picture of water quality. Based on the work of Ojekunle et al. (2016), the RI represents different classification categories. The MI is a general index applied for different types of water uses, e.g., river waters (Bakan et al. 2010; Khoshnam et al. 2017). The RI was proposed by Håkanson (1980) for sediments and aims to assess PTE’s characteristics and environmental behavior for basins/lakes. Although, according to more recent studies, several authors have applied similar indexes to evaluate the potential ecological risk for water (e.g., Karunanidhi et al. 2022).

The first is calculated using the following equation:

1 $${\text{MI}}=\sum\nolimits_{i=1}^{N}{C}_{i}/{(MAV)}_{i}$$

According to Tamasi and Cini (2004) and Withanachchi et al. (2018), ${C}_{i}$ is each PTE concentration in each sample, and MAV is the standard maximum allowed concentration, as defined by the European and Portuguese legal framework for water quality (Decree Law No. 236/98).

The second one was achieved through:

2 $$RI=\sum\nolimits_{i=1}^{8}{Er}^{i}=\sum\nolimits_{i=1}^{8}{T}_{r}^{i}.{C}_{f}^{i}$$

where ${T}_{r}^{i}$ is the toxic response factor (Håkanson 1980) and the ${C}_{f}^{i}$ is the contamination coefficient of a specific PTE, calculated by measured value obtained in the field sample, divided by the reference value, which is the MAV $({C}_{f}^{i}={C}_{sl}^{i}/{MAV}_{n}^{i})$.

However, because the index results may be out of adjustment for different types of water, this investigation proposes an adaptation and addition of new classes of potential ecological risk and Metal Index (MI). This new organization is presented in Table 1.

**Table 1:** Different contamination degrees and classes are presented concerning MI and RI values, respectively. *A new approach proposal (adapted from Dong et al. 2007; Jiao et al. 2012)

Contamination degree	Classes
< 150, low	1
> 150 < 300, moderate	2
> 300 < 600, strong	3
> 600 < 5000, very strong*	4*
> 5000, extremely strong risk*	5*

## Results and discussion

### Hydrochemistry in the mining area

**Fig. 4:**
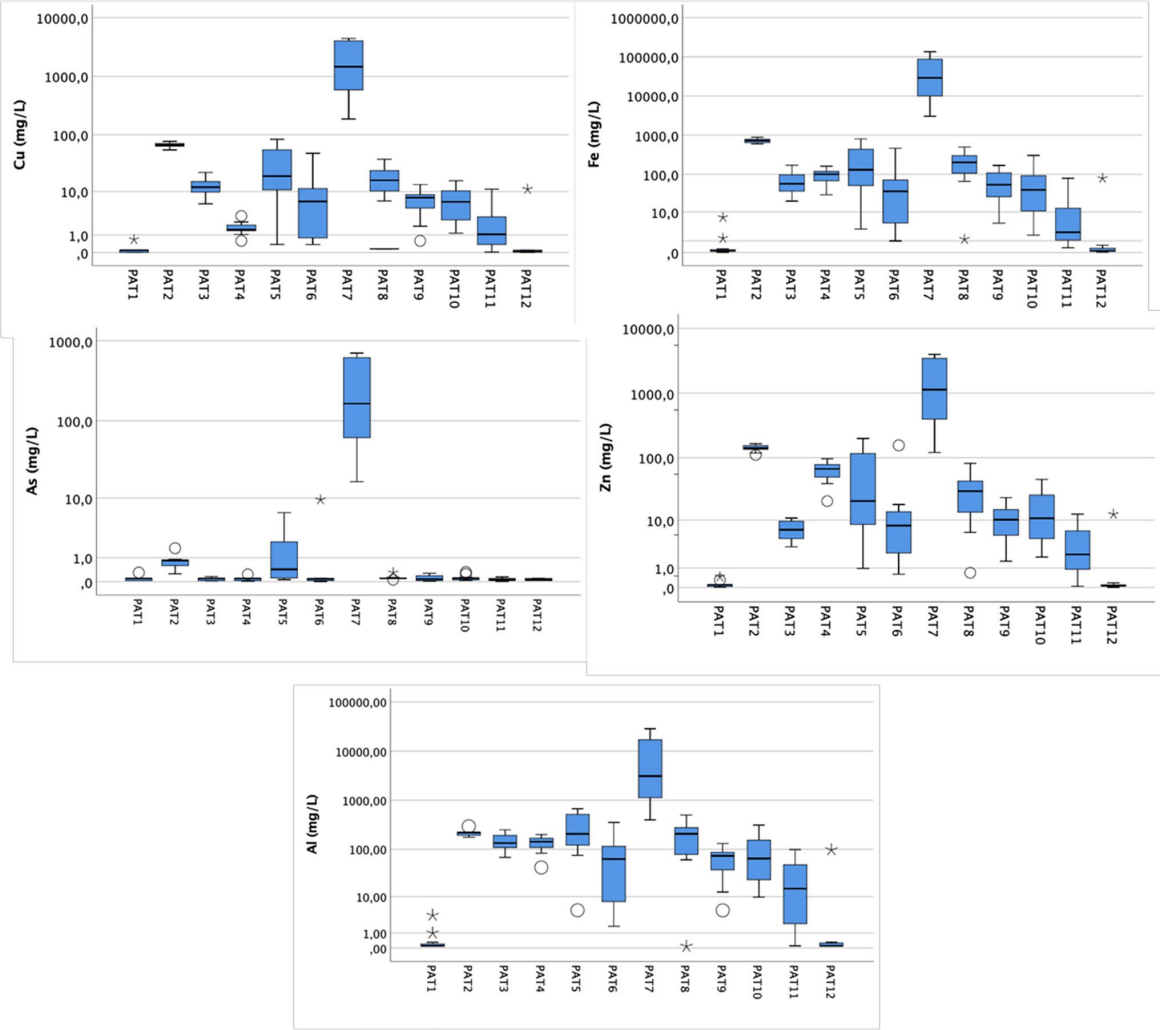
Box and whisker plots of selected PTE analyzed during a completed hydrological (12 campaigns) in the respective sampled points. The box plot displays the interquartile range with the median represented by the line inside the box. The whiskers are lines extending from the box to the highest and lowest, excluding outliers, representing extreme (star symbol) or mild outliers (circle symbol)

Table 2 presents the statistical summary of in situ parameters and sulfate. The box and whisker plots regarding the PTE selected are shown in Fig. 4. Both were analyzed monthly in the hydrological year of 2016/2017 and are crucial AMD indicators.

**Table 2:** Statistical summary of expeditious parameters and sulfate, analyzed over 12 campaigns. Avg = average; Min = minimum; Max = maximum

Samples		pH	EC (μS/cm)	Eh (mV)	SO_4_ (mg/L)
PAT1	Avg	7.1	247	217	8.6
	Min	5.2	28	49	2.2
	Max	8.1	308	352	25.3
PAT2	Avg	2.6	7297	552	5873
	Min	2.4	741	506	4742
	Max	3.0	8850	615	6699
PAT3	Avg	2.7	2877	517	1608
	Min	2.3	228	498	662
	Max	3.5	4378	543	2761
PAT4	Avg	2.7	2655	555	1728
	Min	2.3	310	487	552
	Max	3.0	4230	605	2600
PAT5	Avg	2.5	4035	538	3155
	Min	2.1	251	272	866
	Max	2.8	6943	619	6565
PAT6	Avg	3.0	1735	480	1002
	Min	2.6	117	216	68.2
	Max	4.0	4320	545	2955
PAT7	Avg	1.6	16,132	514	157,230
	Min	0.4	1252	447	10,124
	Max	2.5	27,300	657	410,601
PAT8	Avg	2.8	3393	506	2498
	Min	2.2	182	288	58.5
	Max	4.7	7320	576	5732
PAT9	Avg	2.9	1729	515	836
	Min	2.4	204	374	110
	Max	3.5	3186	566	1642
PAT10	Avg	2.9	3200	470	2055
	Min	2.4	186	242	151
	Max	3.7	8598	561	6753
PAT11	Avg	4.0	741	371	301
	Min	2.9	169	84.8	58.4
	Max	6.3	1527	516	790
PAT12	Avg	6.5	249	285	34.1
	Min	5.3	26.5	114	24.8
	Max	7.9	503	391	48.2

The highest pH value was detected in the dam upstream of the mining area, PAT1, with 8.13. In the opposite situation, there is PAT7, with the lowest pH recorded (0.4). EC is also highlighted in PAT7, as it has the highest registered value, with 27,300 μS/cm, in May 2017. Comparing specifically with other sites in the IPB, González et al. (2020) refer to EC values of around 11,000 μS/cm in the Tharsis mines. So, in the present study, São Domingos (PAT7) presents an even superior EC. As expected, higher values for Eh (500–600 mV) are observed in environments that are presumed to be more oxidizing, associated with the evolution of AMD. This parameter generally behaves similarly to the EC along the sampled path. PAT7, PAT2, and PAT5 exhibit higher sulfate concentrations (410,601; 6,699; and 6,565 mg/L, respectively).

The results of PTE demonstrate that PAT7 and PAT2, followed by PAT5, are the most contaminated sites, and Fe and Al have higher concentrations. The results obtained for Al can be explained by the abundant dissolution of felsic host rocks, increased by the medium's strong acidity, as suggested by Soyol-Erdene et al. (2018)On the other hand, high Fe concentrations may be related to the paragenesis of the study area, very rich in pyrite, whose availability is associated with AMD. For example, Fe presents a concentration of 134,000 mg/L in PAT7. These values agree with others registered in different parts of the world, corresponding to extreme AMD pollution cases. According to Giloteaux et al. (2013), Carnoulés, in France, also exhibits extreme sulfate concentrations, up to 30,000 mg/L, and low values for pH (down to 1.2). A paradigmatic example is the Richmond Mine at Iron Mountain, which presents 111,000 mg/L of Fe, 23,500 mg/L of Zn, 340 mg/L of As, and 760,000 mg/L of sulfate (Nordstrom and Alpers 1999). According to Migaszewski et al. (2014), Wisniówka tailing pile pools in Poland show 66,000 mg/L of Fe, 1500 mg/L of As, pH values of 1.2, and 330,000 mg/L of sulfate concentrations.

The most contaminated samples revealed higher range values for almost all the PTEs analyzed, indicating a stronger seasonality impact (PAT5 and PAT7). According to Zhou et al. (2022), Cu and As are more associated with the precipitation, and they might decrease abruptly when the precipitation is above 4.68 mm. However, the results referring to PAT2 (also a very contaminated site) do not reveal the same behavior, that is, a large variation in the concentration of these elements throughout the hydrological year. This event may be because PAT2 is a pit lake whose hydrochemical component appears to remain more stable (Gomes and Valente 2019). Despite presenting with a lower degree of contamination, the remaining points, such as PAT6, PAT10, and PAT11, also appear to reflect some variation in the Cu, Fe, Zn, and Al concentrations. One factor contributing to these changes is that these sampling points may vary from lotic to lentic environments in the dry season. So, with the decrease in precipitation (in the driest months), these points adopt lentic characteristics. Thus, seasonality appears to have a higher impact on the concentration of PTE, revealed in bigger or less water contamination and inevitably in its quality. PAT1 and PAT12 do not show ample variation throughout the hydrological year. These sampling points reveal low concentrations of analyzed PTE.

### Trends of fluvial system-Ficklin diagram

**Fig. 5:**
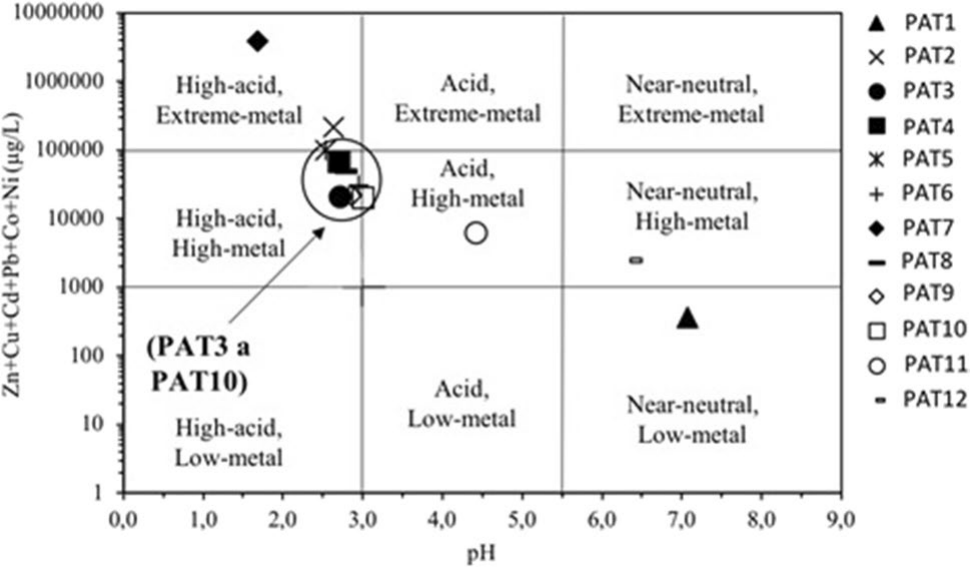
Ficklin diagram (1992) showing different classifications for water sampled at the São Domingos mine

Ficklin diagram (Ficklin et al. 1992), in Fig. 5, reveals the result of 12 sampling points projected according to the sum of metals and their respective pH. It is possible to observe a clear distinction among samples. Thus, PAT7 and PAT1 are at opposite extremes, with “high-acid, extreme-metal” classifications and “near-neutral, low-metal”, respectively. In the same range as PAT7, however, with a higher pH and lower concentration of metals, is PAT2. These results are in accordance with the obtained by Gonzalez (2020) for Tharsis mine. In the previous study, the projection in the Ficklin diagram revealed that samples are mostly classified as high acid-extreme metal and high acid-high metal, like PAT2. However, PAT7 exhibits more extreme values in the São Domingos mine. Another study (Sarmiento et al. 2018) focuses on a lagoon designated by “radical point” and located in a small mining leachate dam (IPB, Spanish side, in Cobica River watershed), revealing a negative pH, and that seems similar to PAT7, studied here. PAT7 seems to have extreme contamination characteristics, being an acidic lake in the industrial area of the complex. The more considerable degree of contamination seems to be strongly associated with the presence of the most reactive wastes, such as accumulations of washed ore, that is, materials highly enriched in fine-grained pyrite disposed of, for example, around the most acidic lagoon — PAT7 (e.g., Sarmineto et al. 2018; Tavares et al. 2009). The digital surface model obtained by Gomes et al. (2021) indicates the influence of drainage pathways on water chemistry in this area. This information confirms the direct relationship between the nature of the materials in the surrounding area and the degree of impact on the water environment. Cordeiro et al. (2017) identified the waste dumps and landfills with the highest contamination potential. These are located in the North sector (near the pit lake) and in the industrial area of Achada do Gamo (PAT7), in line with the results obtained in the present study. The very fine accumulations of washed ore had high reactivity index (pH paste < 3.0). So, according to the results, and similar to what was investigated by other authors (Pérez-López et al. 2008; Sarmiento et al. 2018; Grande et al. 2015), the finest mining wastes are responsible for more harmful effects on the environment. PAT11 and PAT12 are arranged separately in the “acid, high-metal” and “near-neutral, high-metal” ranges. All other samples are grouped in the same classification: “high-acid, high-metal,” emphasizing the PAT5 point, located at the interface with the most concentrated range of metals and the lowest pH. The results can be related to the disposition, proximity, and quantity of waste in mining areas. PAT2 is highly contaminated in this case due to low pH and extreme metal concentration. Additionally, surrounding wastes (reactive slags) can contribute to runoff phenomena in the pit lake. After points PAT3, PAT4, PAT5, and PAT6, there appears to be a tendency towards a reduction in contamination along the sampling line. In addition to reducing the amount of waste disposed along the watercourse and its reactivity, another aspect that can contribute to the reduction of contamination after point PAT8 is the confluence of the Mosteirão River. This clean stream without metallic contamination promotes an increase in the hydrological load, which in turn is reflected in the dilution effect.

Furthermore, from PAT11 onwards, there tends to be decreased contamination, mainly because of dilution and higher distance from the waste accumulation. Also, mineralogical controls related to the precipitation of jarosite and other secondary phases contribute to this attenuation, as Alpers et al. (1994) referred to. PAT12, water for human consumption, was revealed to be a point of clean water, as indicated by lower concentrations of the selected elements.

### Seasonal variability and environmental quality

**Fig. 6:**
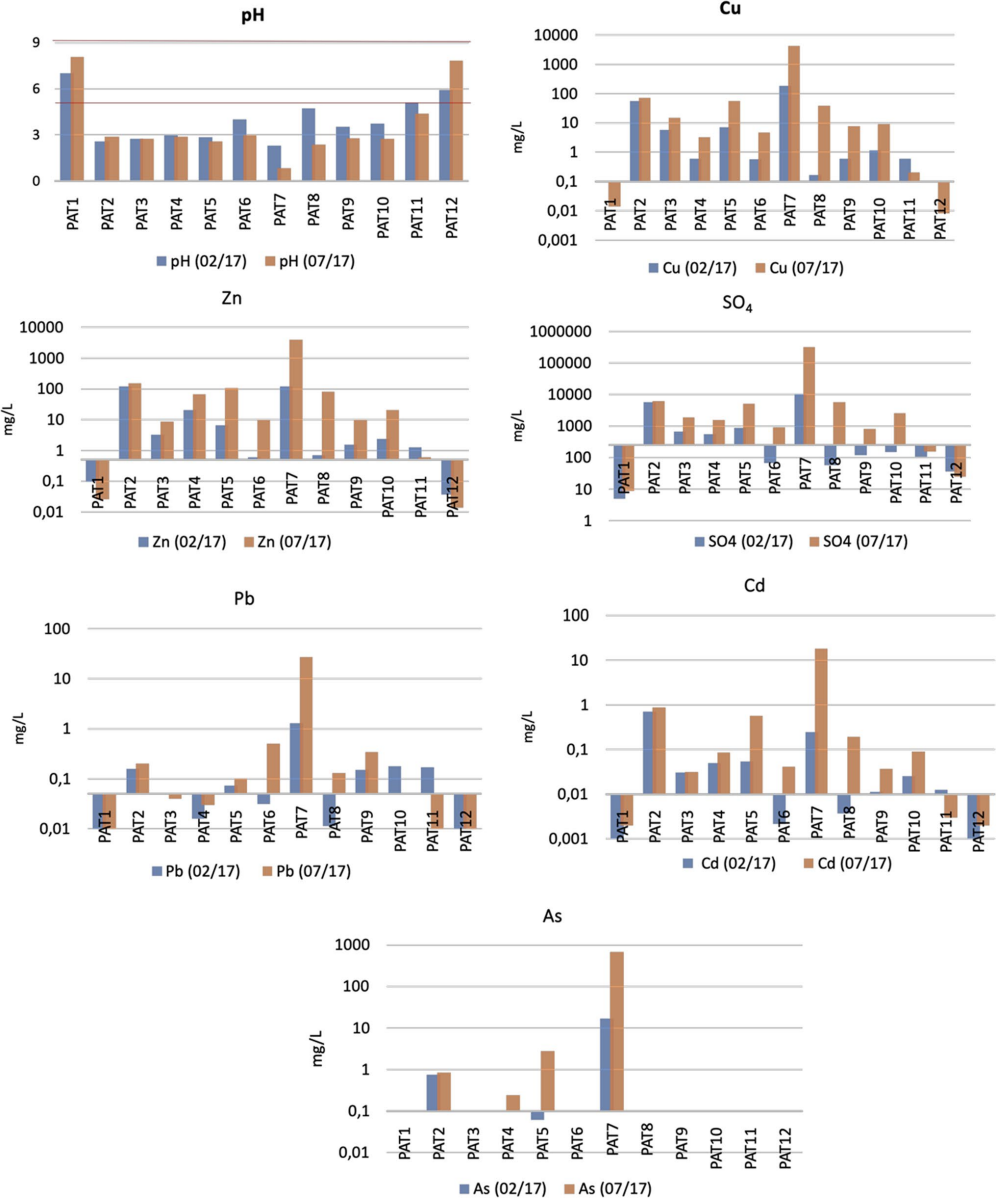
Hydrochemical variations referring to the rainiest month (02/17) and the driest month (07/17), considering the quality environmental objectives for surface waters (Decree Law No. 236/98, of August 1st – Annex XXI). The *x*-axis represents each MAV value: pH (5–7); Cu = 0.1; Zn = 0.5; S0_4_ = 250; Pb = 0.05; As = 0.1; Cd = 0.01

Figure 6 reveals the maximum allowed values (MAV), considered as standards for environmental objectives of quality for surface waters (pH, Cu, Zn, SO_4_, Pb, As, and Cd) (Decree Law No. 236/98, of August 1) in different periods: the wettest (02/17) and driest month (07/17). Only PAT1 and PAT12 proved to comply with MAV (water dam for recreational use and water dam for human consumption, respectively). As regards the other points, it is possible to notice that all elements suffered notable variations, with higher concentrations in the dry month (July 2017 campaign). The MVA was largely exceeded. The pH value at PAT7 is exceptional compared to the other analyzed data, with a value of 0.84 in the July campaign, well below the range presented as admissible (between 5 and 9). Despite not reporting extreme events in their study, Sarmiento et al. (2018) have found, between October 2003 and January 2007, negative pH values on the other side of the border, also in a lagoon of an old IPB mine. Such results are justified by the low dilution in the context of lesser water availability, leading to the intense water–rock interaction processes (Cánovas et al. 2007).

Nevertheless, some elements showed minimum concentrations in the dry period, probably due to the intense precipitation of Fe oxyhydroxysulfate and sulfates that incorporated some elements in their structure (González et al. 2020). These lower concentrations occur for Pb and Cd in PAT11. According to Farkas et al. (2007); Duodu et al. 2016), after some metals are introduced into the aquatic ecosystem, most can be attached to fine-grained particles and accumulate in bottom sediments through settling. Even when water quality criteria are not exceeded, these substances can harm biological systems (Bibi et al. 2007). In this regard, during field sampling, it was possible to visually confirm the presence of ochre precipitates, which were more intense at the end of the channel, specifically in locations PAT10 and PAT11. These precipitates formed long, thick pastes deposited on the watercourse bed. Furthermore, Rao et al. (2021) reported that the metals with the highest potential eco-risk were Pb and Cd. These were retained in the sediments, presenting high concentrations. Also, concerning Pb, Gomes et al. (2021) made it possible to verify in their study of clusters an intrinsic association of this element with algae in the aquatic environment. These algal mats could be accumulating these metals in their cells. Moreover, Yu and Wang (2004) and Yuan et al. (2015) found that eutrophication can stimulate the absorption and accumulation of toxic metals in freshwater phytoplankton cells, which can affect their mobility in the aquatic ecosystem.

Several authors have reported for decades (e.g., Johnson and Hallberg 2005; Gomes et al. 2018) that AMD from abandoned mines can be the main limiting factor for water use in the river basins in which they are located, as well as the primary source of ecological degradation of river systems. Thus, the São Domingos and Mosterirão streams are outside the stipulated environmental quality objectives. It should be noted that according to the EU Commission (2000), the main goal of the EU Water Framework Directive (WFD) is to achieve good ecological and chemical quality for all European rivers. In this sense, the results obtained for this mining site are clearly out of the scope of the legal framework and the United Nations Sustainability Development Goals (SDG-UN, Agenda, 2030).

### Drought episodes: potential ecological risk

PTE’s implications for the ecosystem were analyzed in the driest conditions, as they present more extreme values and, therefore, higher risk. In this way, Table 3 exposes the results of the MI and RI.

**Table 3:** The metal index (MI) and the potential ecological risk index (RI) for sampled sites in the driest month (07/17). Five classes and contamination degrees are presented concerning RI values obtained (adapted from (Dong et al. 2007; Jiao et al. 2012). *A new approach proposal

Driest month	MI	RI	Contamination degree	Class
PAT1	1.392	11.552	< 150, low	1
PAT2	1143.1	6550	> 5000, extremely strong risk*	5*
PAT3	175.12	854.08	> 600 < 5.000, very strong*	4*
PAT4	186.26	578.3	> 300 < 600, strong	3
PAT5	874.82	5047.2	> 5000, extremely strong risk*	5*
PAT6	82.72	427.9	> 300 < 600, strong	3
PAT7	59,162.4	341,594	> 5000, extremely strong risk*	5*
PAT8	580.38	2675.8	> 600 < 5000, very strong*	4*
PAT9	110.04	555.62	> 300 < 600, strong	3
PAT10	145.92	764.9	> 600 < 5000, very strong*	4*
PAT11	4.662	25.262	< 150, low	1
PAT12	1.308	11.228	< 150, low	1

The analysis indicates that PAT1, PAT11, and PAT12 have low potential ecological risk. PAT4, PAT6, and PAT9 belong to class 3, exhibiting strong risk. PAT3, PAT8, and PAT10 reveal one of the proposed new classes (class 4), evidencing very strong risk. As revealed throughout the study, PAT2, PAT5, and PAT7 are classified in the highest proposed class (> 5000), indicating potential extremely strong ecological risk. In this way, it is possible to order the sampling points by groups, depending on the ecological risk of contamination: PAT1, PAT11, PAT12 < PAT4, PAT6, PAT9 < PAT3, PAT8, PAT10 < PAT2, PAT5, PAT7. No samples are in the moderate class (> 150 < 300). The index thus reveals extreme classification values. According to Bakan et al. (2010), the results of MI are even more pessimistic, indicating that all sampled points are representative of a warning limit (MI > 1). According to Batty et al. (2010) and Reyes-Becerril et al. (2019), elements such as Cu, Zn, Pb, As, or Cd are directly toxic to the metabolism of aquatic organisms, and their release poses an environmental and health threat.

## Conclusion

The AMD-studied waters have very high EC, low pH, and very high concentrations of dissolved PTE. They are classified mostly as high-acid, extreme-metal, and high-acid, high-metal. A seasonal pattern in AMD hydrochemistry can be observed: the rainy month has lower PTE. In contrast, the driest month is the most contaminated, revealing the impact of extreme drought episodes on the water medium. In addition to not complying with the environmental objectives in the legislation, the water quality is not in line with the United Nations Sustainability Development Goals. Only PAT1 (recreation dam) and PAT12 (dam for human consumption) agree with the minimum quality environmental objectives for surface water in both studied months: dry and rainy. However, the MI reveals that all samples are representative of a warning limit (> 1). The potential ecological risk shows extreme classes, and the risks presented in this study may have major implications in the future, considering the current water scarcity scenario. According to the preliminary document of the Intergovernmental Panel on Climate Change (IPCC, ONU), published in 2021, the region is in a “hot spot,” being subject to extreme climate changes, implying a strong probability of heat waves and extreme risk of drought. Thus, the new classifications can alert competent authorities to implement preventive and even remedial measures in line with different risks determined.
